# Supplementary material for: Quantum effect-based flexible and transparent pressure sensors with ultrahigh sensitivity and sensing density
Source: Nat Commun. 2020 Jul 15;11:3529. doi: 10.1038/s41467-020-17298-y (PMC7363923; doi:10.1038/s41467-020-17298-y)
Supplement: Supplementary file 1 — Supplementary Information [file 41467_2020_17298_MOESM1_ESM.pdf]

Supplementary Information

Quantum effect-based pressure sensor achieving ultrahigh sensitivity and sensing density

Shi et al.

Supplementary Figures

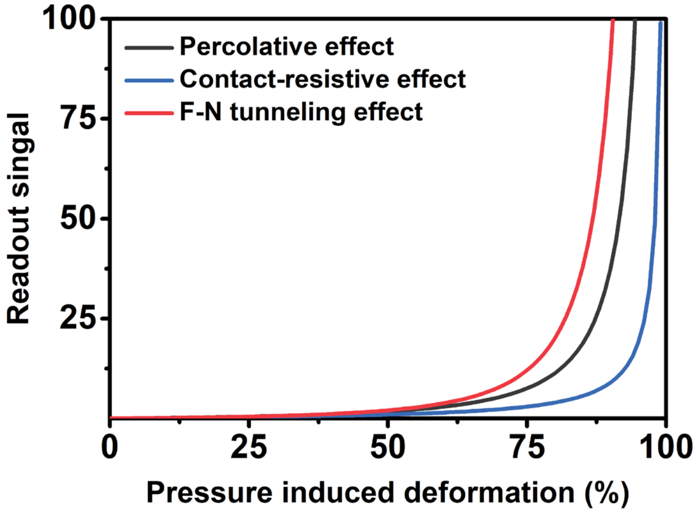

Supplementary Figure 1. Comparison curves of the three mechanisms. Readout signals as a function of pressure-induced deformation based on three transduction mechanisms were calculated and shown by the red curve (F-N tunnelling), black curve (Percolative) and blue curve (Contact-resistive), respectively.

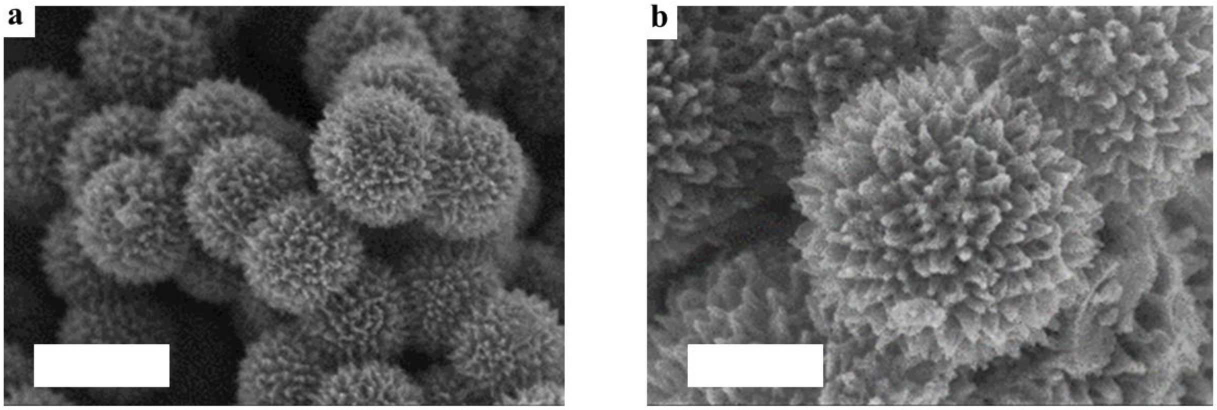

Supplementary Figure 2. SEM images of the unannealed polyaniline spheres. Scale bar, **a,** 700 nm. **b,** 300 nm.

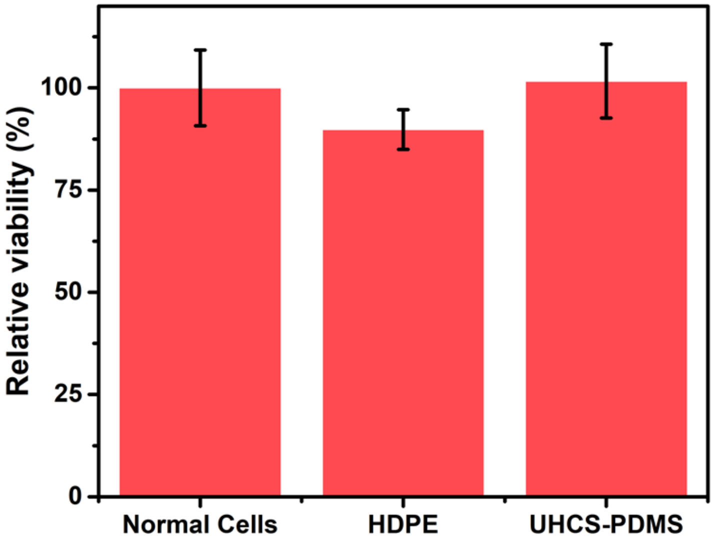

Supplementary Figure 3. Cytotoxicity assay using spectrophotometry. Cytotoxicity of UHCS-PDMS, HDPE (as a non-toxic material), and normal cells (as blank control) in NIH 3T3 cells. Relative cell viability (%) = mean OD of experiment group/ mean OD of blank control group ×100%. The error bars represent one standard deviation.

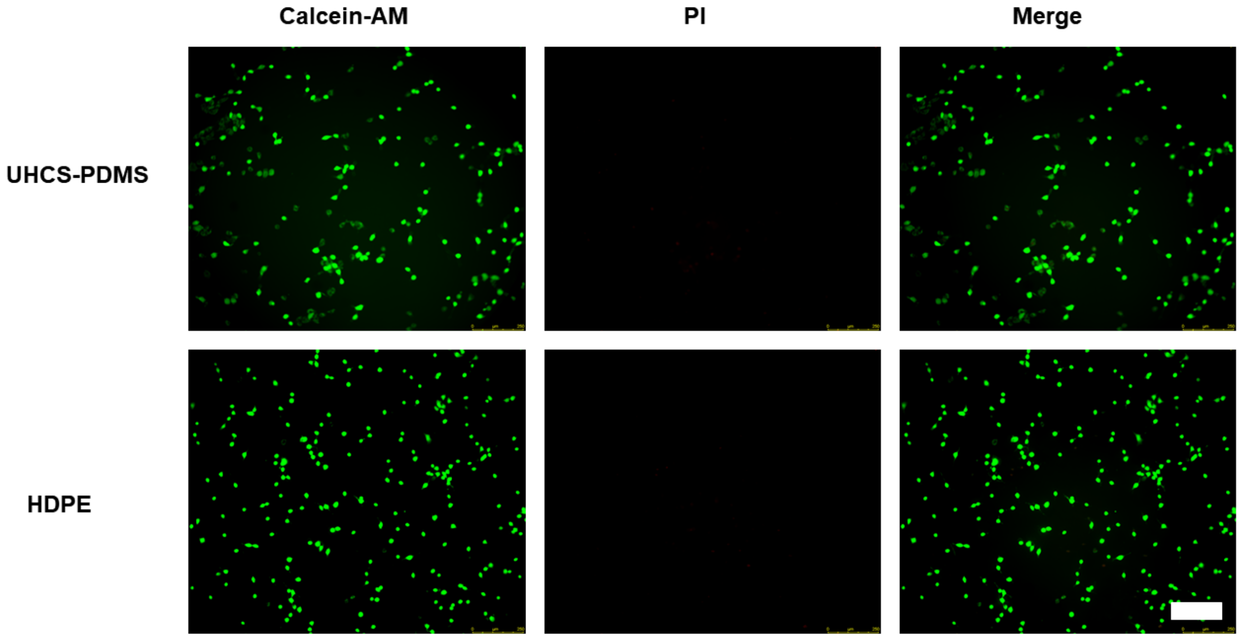

Supplementary Figure 4. Cytotoxicity assay using fluorescence microscopy. Calcein-AM/PI staining of NIH 3T3 cells incubated with UHCS-PDMS and HDPE, respectively. The live cells were stained green, and the dead cells were stained red, scale bar 250 μm.

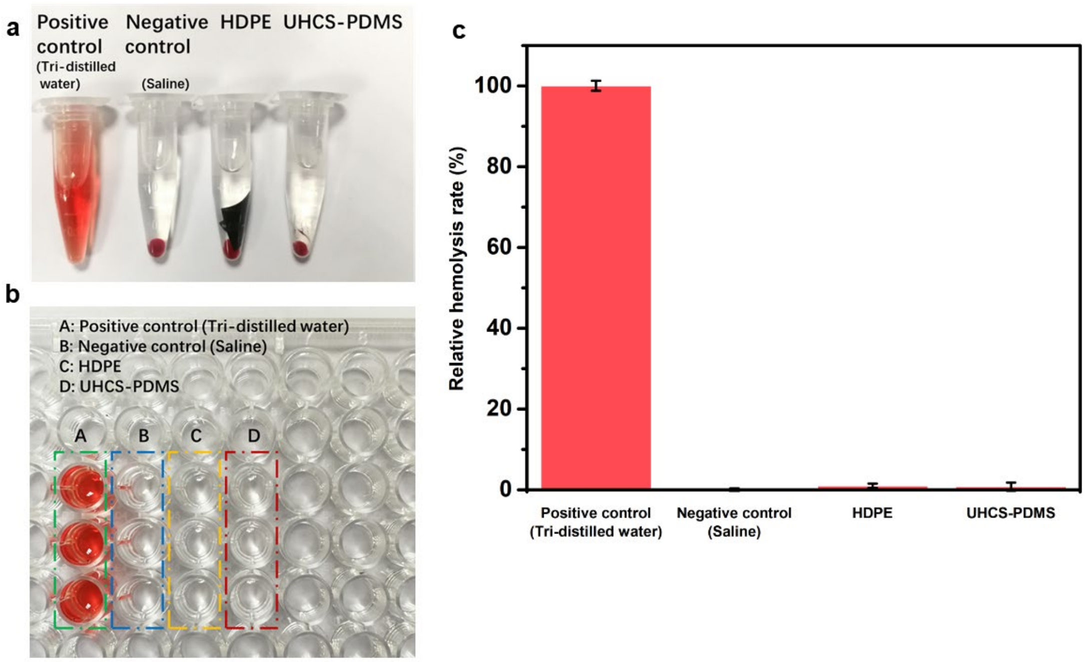

Supplementary Figure 5. Blood compatibility assay. Hemolysis of UHCS-PDMS, HDPE (as a non-toxic material), tri-distilled water (as positive control), and saline (as negative control), after 2 h incubation with red blood cell suspension at 37 °C. **a,** the tubes after incubation and centrifugation. **b,** the supernatants for absorbance determination. **c.** the histogram shows the results of each groups. The error bars represent one standard deviation.

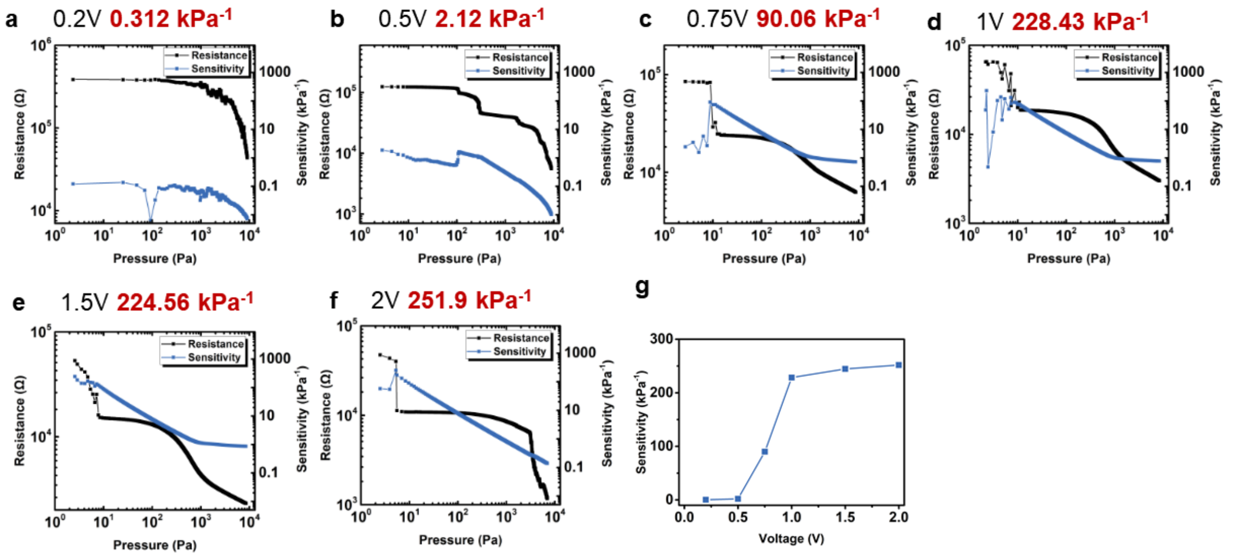

Supplementary Figure 6. Pressure sensing behaviour under different testing voltage. a, When voltage is 0.2 V, the highest sensitivity is 0.312 kPa^{-1}. **b,** When voltage is 0.5 V, the highest sensitivity is 2.12 kPa^{-1}. **c,** When voltage is 0.75 V, the highest sensitivity is 90.06 kPa^{-1}. **d,** When voltage is 1 V, the highest sensitivity is 228.43 kPa^{-1}. **e,** When voltage is 1.5 V, the highest sensitivity is 224.56 kPa^{-1}. **f,** When voltage is 2 V, the highest sensitivity is 251.9 kPa^{-1}. **g,** The relationship between voltage and sensitivity.

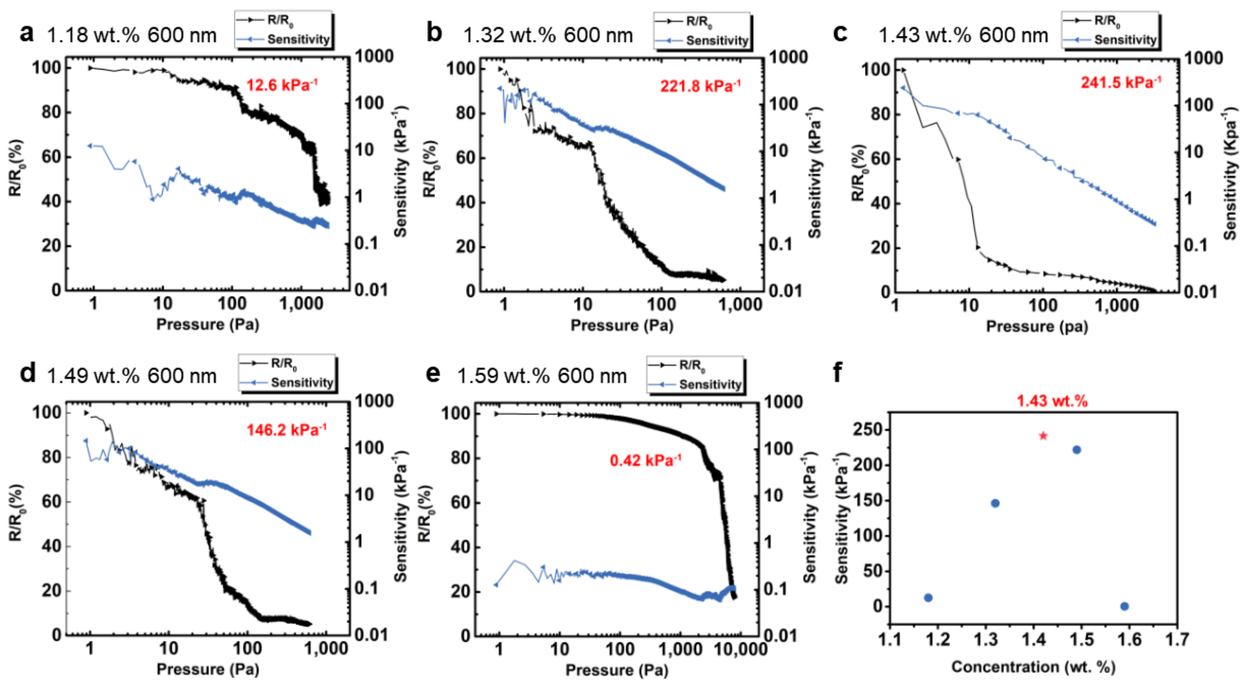

Supplementary Figure 7. Pressure sensing behaviour of different UHCS contents. **a,** When c_{UHCS} is 1.18 wt.%, the highest sensitivity is 12.6 kPa^{-1}. **b,** When c_{UHCS} is 1.32 wt.%, the highest sensitivity is 146.2 kPa^{-1}. **c,** When c_{UHCS} is 1.43 wt.%, the highest sensitivity is 241.5 kPa^{-1}. **d,** When c_{UHCS} is 1.49 wt.%, the highest sensitivity is 221.8 kPa^{-1}. **e,** When c_{UHCS} is 1.59 wt.%, the highest sensitivity is 0.42 kPa^{-1}. **f,** The relationship between UHCS content and sensitivity.

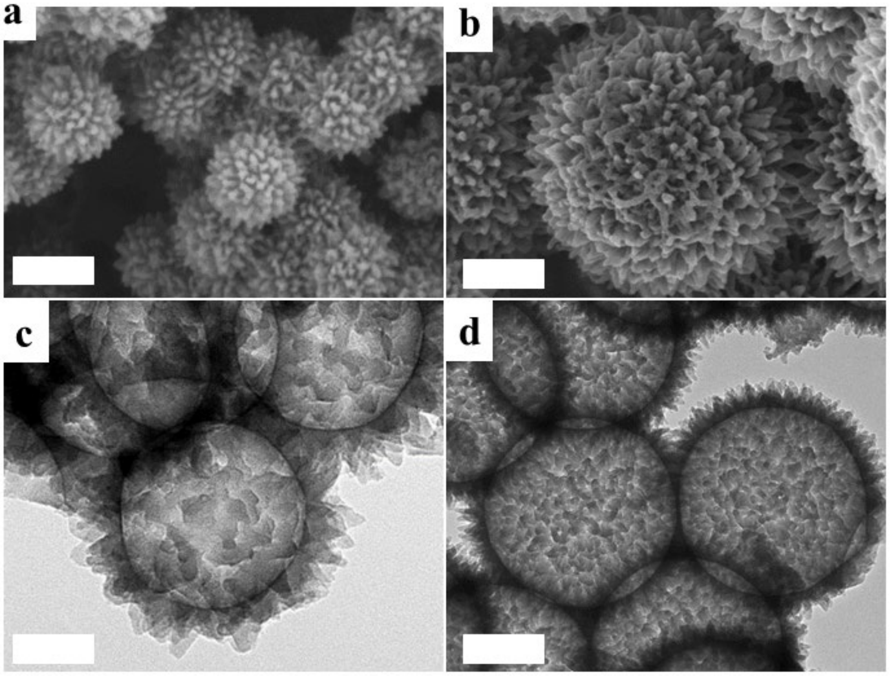

Supplementary Figure 8. SEM and TEM images of UHCS with 300 nm and 900 nm in diameter. a, SEM image of 300 nm UHCS, scale bar, 300 nm. **b,** SEM image of 900 nm UHCS, scale bar, 250 nm. **c,** TEM image of 300 nm UHCS, scale bar, 120 nm. **d,** TEM image of 900 nm UHCS, scale bar, 350 nm.

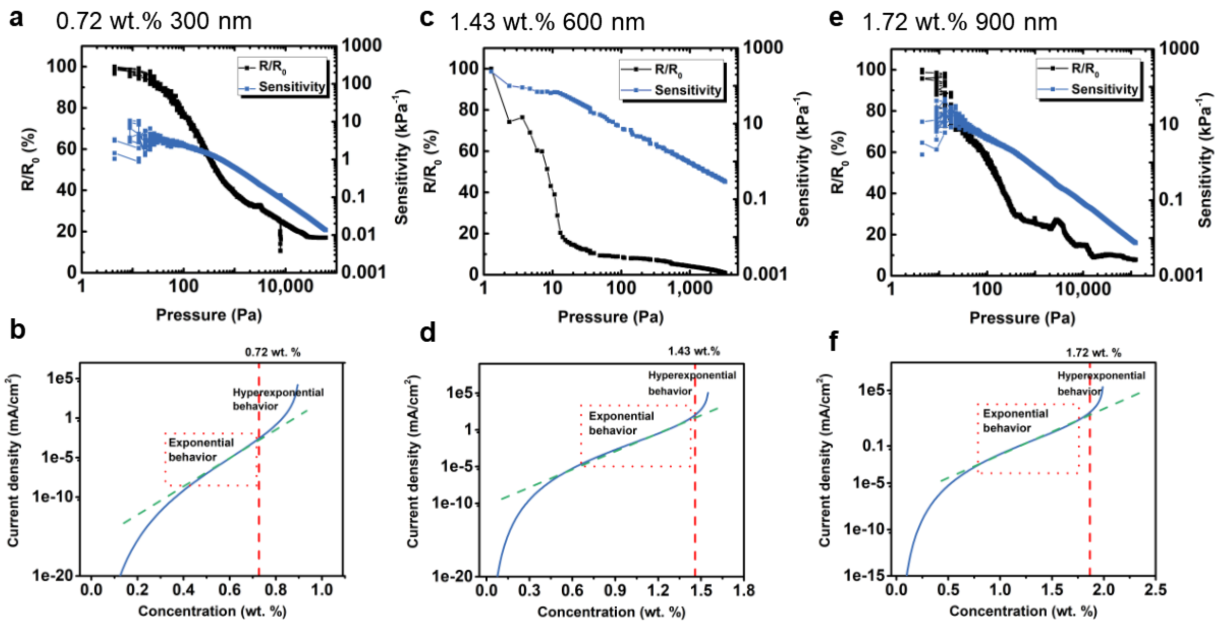

Supplementary Figure 9. Resistance response to pressure with different UHCS diameters. Since the change in UHCS diameter will change the optimum concentration of UHCS for pressure sensing, the current density as a function of UHCS concentration is plotted for each UHCS. The optimum c_{UHCS} is selected when it is just at the transition from exponential behaviour to hyperexponential behaviour of the curve. **a,** When d_{UHCS}= 300 nm, the optimum c_{UHCS} determined from **b** is 0.72 wt.%, and its highest tested sensitivity is 10.66kPa^{-1}. **c,** When d_{UHCS}= 600 nm, the optimum c_{UHCS} determined from **d** is 1.43 wt.%, its highest tested sensitivity is 241.5 kPa^{-1}. **e,** When d_{UHCS}= 900 nm, the optimum c_{UHCS} determined from **f** is 1.72 wt.%, its highest tested sensitivity is 47.6 kPa^{-1}.

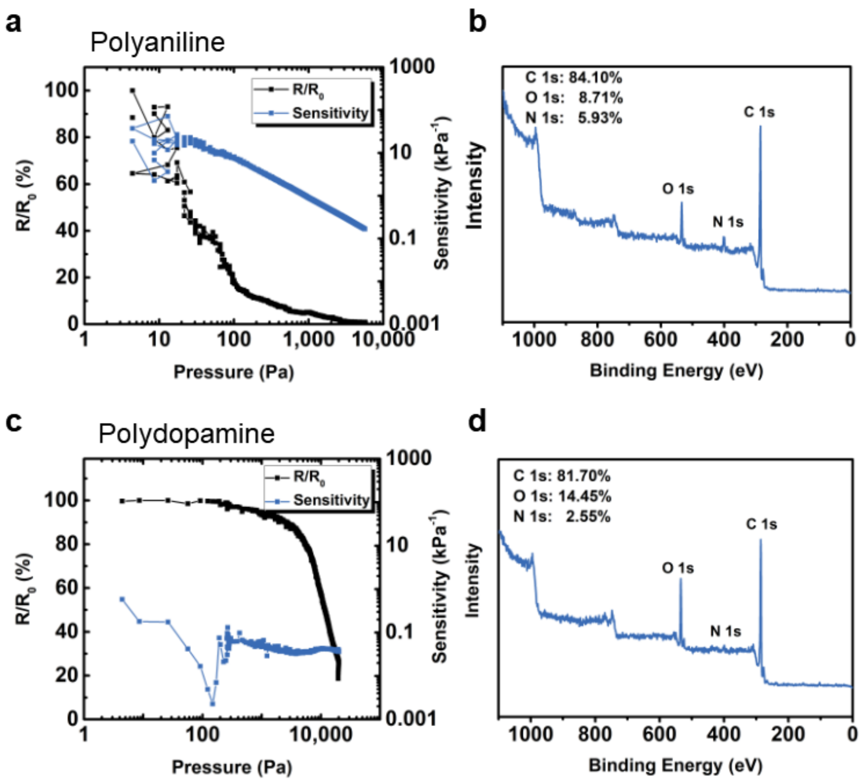

Supplementary Figure 10. Resistance response and pressure sensitivity of the pressure sensor with different precursors. a, Pressure sensor prepared with polyaniline-derived UHCS with the highest sensitivity of 95.1 kPa^{-1}. **b,** XPS survey spectrum of UHCS obtained from polyaniline, the quality fraction of carbon is 84.10 %, and the quality fraction of oxygen is 8.71%. **c,** Pressure sensor prepared with polydopamine-derived UHCS with the highest sensitivity of 0.58 kPa^{-1}. **d,** XPS survey spectrum of UHCS obtained from polydopamine, the quality fraction of carbon is 81.70 %, and the quality fraction of oxygen is 14.45 %.

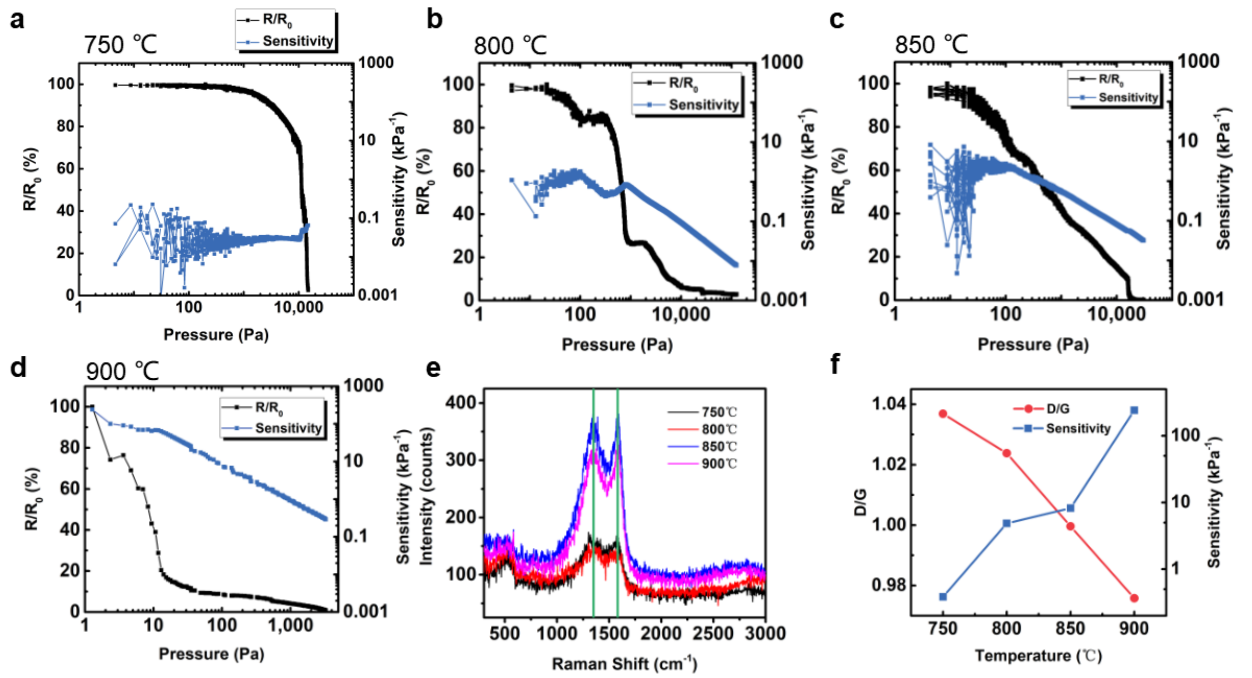

Supplementary Figure 11. Resistance response and pressure sensitivity of the film pressure sensor with different calcination temperatures. a, 750 °C, the highest sensitivity, 0.384 kPa^{-1}. **b,** 800 °C, the highest sensitivity, 4.86 kPa^{-1}. **c,** 850 °C, the highest sensitivity, 8.246 kPa^{-1}. **d,** 900 °C, the highest sensitivity, 241.5 kPa^{-1}. **e,** Raman spectrum of the four UHCS. **f,** The relationship between D/G strength and sensitivity from **e**.

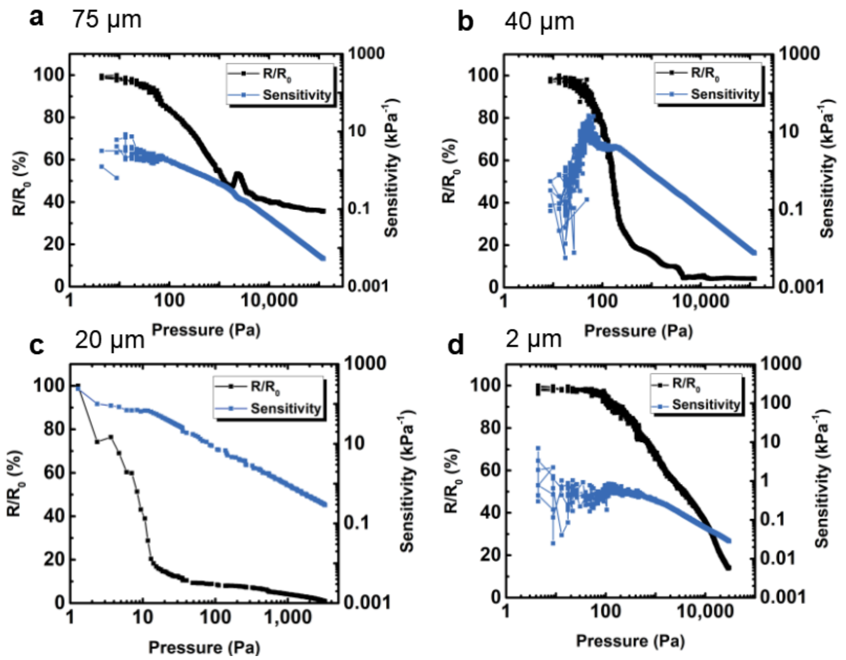

Supplementary Figure 12. Resistance response and pressure sensitivity of the film pressure sensor with different thicknesses. a, 75 µm thick, the highest sensitivity, 8.42 kPa^{-1}. **b,** 40 µm thick, the highest sensitivity, 26.5 kPa^{-1}. **c,** 20 µm thick, the highest sensitivity, 241.5 kPa^{-1}. **d,** 2 µm thick, the highest sensitivity, 4.5 kPa^{-1}.

a b

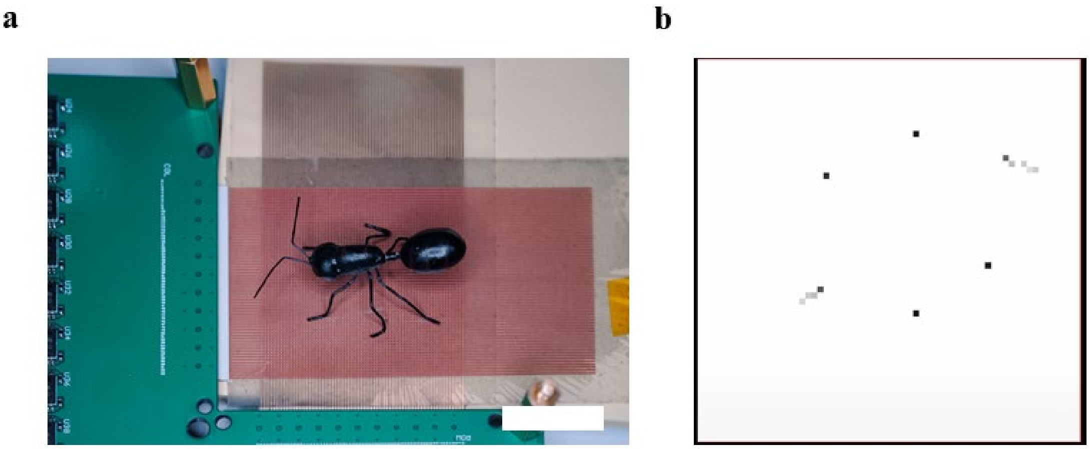

Supplementary Figure 13. Array test of recognising a toy ant. a, Photo of the ant on sensing array, scale bar: 16 mm. **b,** Output image from the readout circuit.

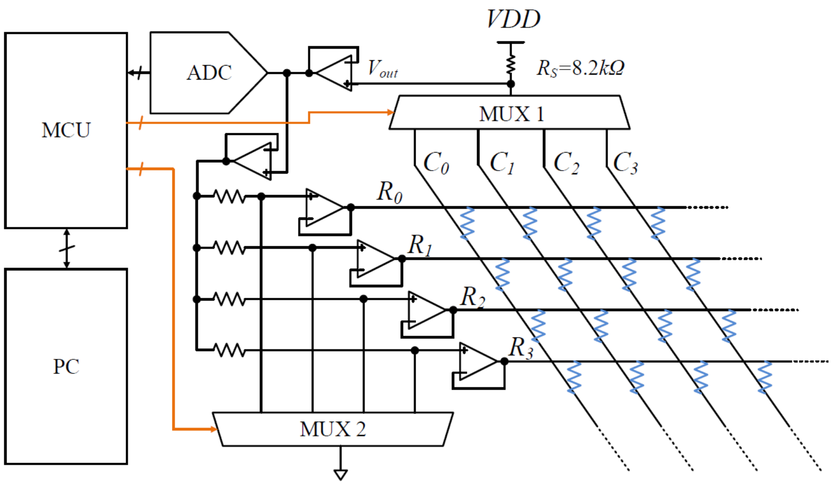

Supplementary Figure 14. Readout circuit of the microcontrollers for the 64 × 64 detection array.

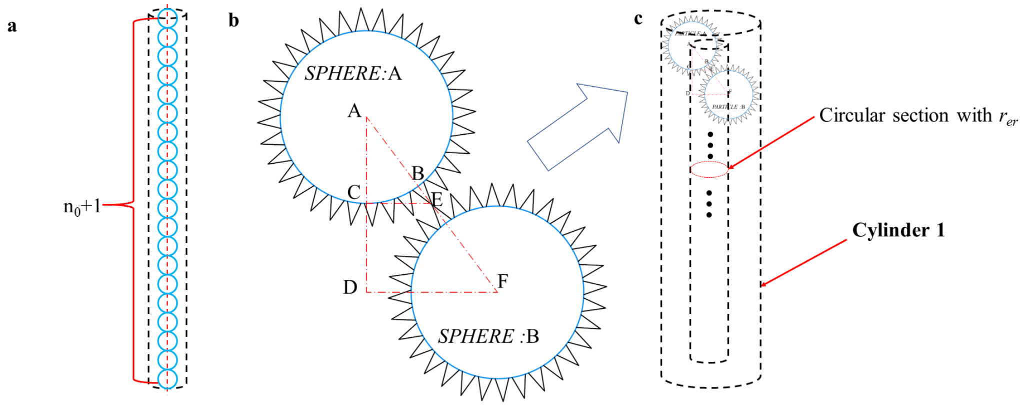

Supplementary Figure 15. The schematic diagram for calculating the effective contact radius.

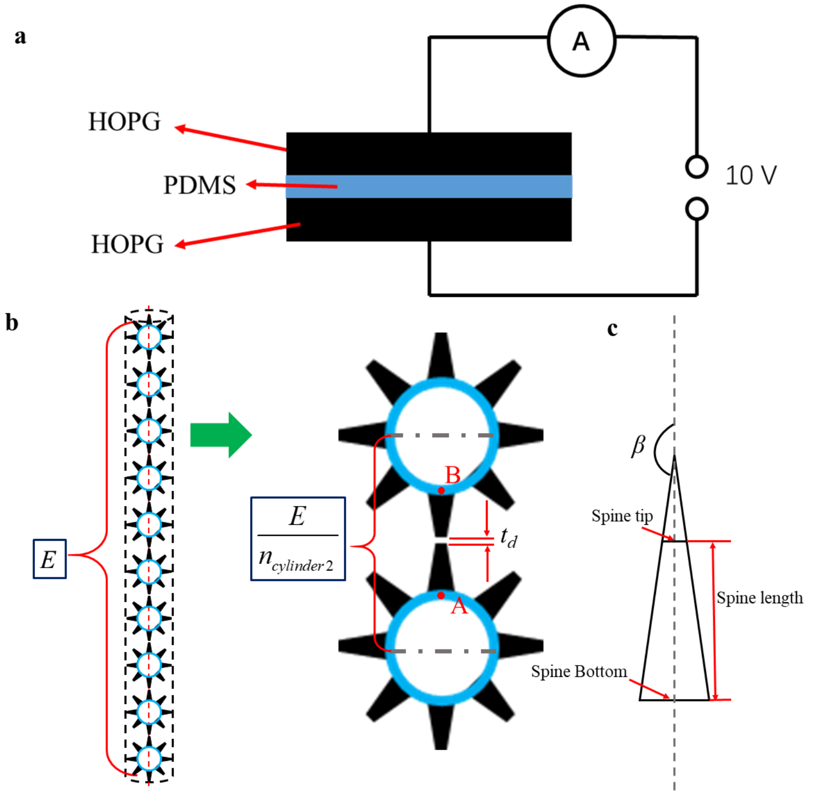

Supplementary Figure 16. Schematic diagram of the measurement to estimate coefficient *A* and *B* in the F-N equation and electric potential calculation method.

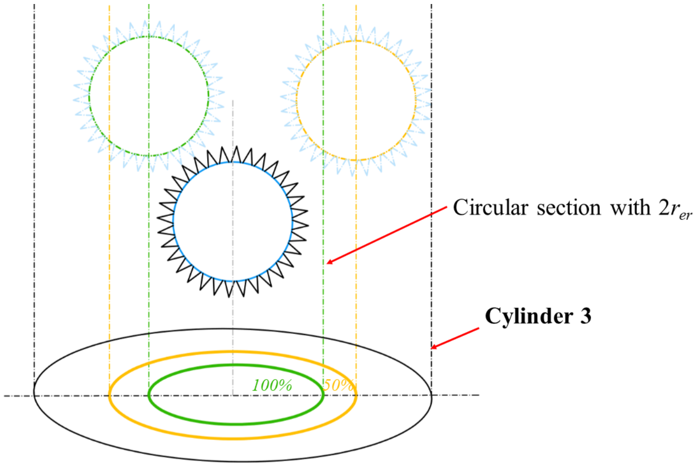

Supplementary Figure 17. Scheme of projection along the height to show the defined area unit of Model 3.

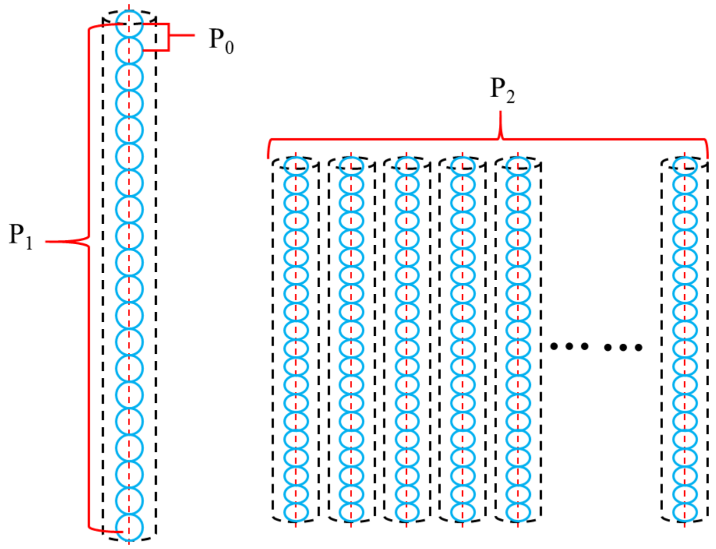

Supplementary Figure 18. Scheme of the *P_0*, *P_1*, and *P_2*.

Supplementary Tables

Supplementary Table 1. Comparison of performances of the pressure sensors.

**Materials**	**Sensitivity** (kPa^{-1})**	**Min detection limit (Pa)**	**Working voltage (V)**	**Response time (ms)**	**Pressure range at sensitivity over 1kPa^{-1} (Pa)**	**Year**
**This work**	**260.3**	**1**	**1**	**30**	**0-800**	**2020**
Honeycomb-like rGO film[1]	161.6	9	0.01~1	-	0- 300	2015
PPy hollow-sphere[2]	133.1	0.8	-	47	0- 200	2014
PEDOT:PSS/PUD[3]	56.8	13	0.2	-	0- 800	2014
Gold film/ PDMS[4]	50.17	10.4	0.01	20	0-200	2015
CNT/G PDMS[5]	19.8	0.6	0.03	16.7	0-300	2017
rGO-paper[6]	17.2	*	-	60	0-1000	2017
PPy/PDMS[7]	17	2	-	-	0-400	2014
rGO foam[8]	15.2	165	-	-	0-300	2014
CNT/PDMS microdome[9]	15.1	0.2	-	40	0-50	2014
PDMS/PEDOT:PSS/Parylene[10]	14.15	0.212	-	-	0-20	2016
Hierarchically graphene /PDMS[11]	8.5	1	1	40	N/A	2016
PDMS microstructure OFET[12]	8.4	*	200	10	N/A	2013
CNWs/PDMS[13]	6.64	*	-	30	N/A	2019
MOFs/PI[14]	6.25	0.73	1	10	N/A	2015
rGO/PDMS microstructure[15]	5.5	1.5	1	0.2	N/A	2014
GMI/air gap[16]	4.4	0.3	1.5	-	N/A	2018
ZnO nanowires[17]	2.1	<3500	1	-	N/A	2013
SWNT/PDMS[18]	1.8	0.6	2	10	N/A	2014
Porous PDMS/air gap[19]	1.5	2.5	-	-	N/A	2014
SWNT/PDMS[20]	1.25	*	10	100	N/A	2013
Au NW/ tissue paper[21]	1.14	13	1.5	17	N/A	2014
Fluorosilicone/air gap[22]	0.91	1.6	-	40	N/A	2014
rGO/molecular pillars[23]	0.82	7	0.2	24	N/A	2019
GO foam[24]	0.8	0.24	-	100	N/A	2017
Micro rubber dielectric[25]	0.55	3	80	<<1000	N/A	2010
pDA-rGO[26]	0.29	*	30	54	N/A	2018

ACC/PAA/alginate mineral hydrogel[27]	0.17	*	3	50	N/A	2017
Graphene/ion gel FET[28]	0.12	5	0.3	500	N/A	2014
Galinstan microchannels PDMS[29]	0.0835	50	0.03	90	N/A	2017
Carbon black/PU sponges[30]	0.068	17	-	20	N/A	2016
Cu NW/PVA[31]	0.036	400	-	-	N/A	2014
SWNT/cotton thread[32]	0.0156	500	-	-	N/A	2016
STENG[33]	0.013	1300	1	200	N/A	2017
Polyamine/AgNPs[34]	0.0067	*	-	-	N/A	2018

* means no related test was shown in the papers, all the minimum pressure has been regarded as 0 Pa.

** The sensitivity was calculated (readout signal change divides external pressure change, $S=\Delta X/\Delta P$) and normalized (the integral of the sensitivity from 0 kPa to infinity should equals to 1, $\int_0^\infty Sdp = 1$) to be comparable.

- means no data were shown in the papers.

Supplementary Table 2. Comparison of performances of pressure sensor arrays.

**Technology**	**Density (cm^{-2})**	**Limitation of high density**	**Year**
UHCS-PDMS (this work)	400	minimum detection area	
Human fingers[35]	70		1979
Human palm[35]	8		1979
Pressure sensor rubber[36]	64	Crosstalk	2013
Stretchable CNT[37]	25	Crosstalk	2011
All-graphene[38]	25	Fabrication limitation	2016
Hemispheres[39]	18	Microstructure	2016
Biodegradable polymer[40]	13	Microstructure	2015
CNT active matrix[41]	8.9	Crosstalk	2015
Organic active matrix[42]	7.3		2005
All-textile[43]	5	Fiber and weaving process	2017
Flexible suspended gate[44]	1.8	Sensor structure	2015
SI-TENG[45]	1.8		2018
Self-healing sensor[46]	1	Enough cross-section area	2012
Organic digital[47]	1		2015

Supplementary Notes

Supplementary Note 1. Relations between Δ*J* and *ε* of the percolation threshold.

According to the effective medium theory (Equation 1), the current density $J(x)$ increases with the mass fraction of conductive fillers x, which reflects the change in thickness when the composite is compressed due to external pressure. The black line in Supplementary Figure 1 shows the relationship between the readout signal and the pressure-induced deformation under the percolation effect.

$$J(x) = J_M (x\text{-}x_c)^t \tag{1}$$

where x is the mass fraction of conductivity fillers, $J(x)$ is the current density of the composite, J_M is the coefficient current density, x_c is the percolation threshold, $t = 1.6$ for the three-dimensional case.

Define ΔJ as the change of current density caused by external pressure, J_0 as the initial current density, thus,

$$J = J_0 + \Delta J \tag{2}$$

Then, define a sample with a cross-section of S_0 and height of d_0, the volume $V_0=d_0\times S_0$. When the pressure applied, d would change Δd, then,

$$d = d_0 - \Delta d \tag{3}$$

Then the mass fraction can be described as follows,

$$x = \frac{m_0}{d_0(1-\frac{\Delta d}{d_0})S_0} \tag{4}$$

Thus, combine Equations 1-4, the relationship between J and Δd is as follows.

$$J = J_{\mathrm{M}}\left(\frac{m_0}{S_0 d_0 (1-\frac{\Delta d}{d_0})} - x_{\mathrm{c}}\right)^t \tag{5}$$

Then ΔJ can be described as follows,

$$\Delta J = J_{\mathrm{M}}\left(\frac{m_0}{S_0 d_0 (1-\frac{\Delta d}{d_0})} - x_{\mathrm{c}}\right)^t - J_0 \tag{6}$$

As strain ε is defined to be $\Delta d/d_0$, the relationship between ΔJ and ε is as follows,

$$\Delta J \propto \left(\frac{1}{1-\varepsilon} - x_{\mathrm{c}}\right)^t \tag{7}$$

It is usually a three-dimensional type in the application, so t=1.6 and x_c can be set as 28.95% when the filler is assumed to be perfect spheres[48].

Supplementary Note 2. Relations between ΔR_{con} and ε of contact resistive.

The relationship between the contact resistance R_{con} and the contact force F is described as equation 8[49] and accordingly, the relationship between the readout signal and pressure-induced deformation is shown by the red line in Supplementary Figure 1.

$$R_{\text{con}} = \frac{k}{(0.102F)^m} \quad (8)$$

where R_{con} is the contact resistance, k is a coefficient related to contact materials, F is the contact force, m is determined by the contact form (empirical studies have shown that when the contact form is point-type, m= 0.5; when it is line-type, m is between 0.5-1, approximately 0.7; when the contact form is face-type, m= 1).

While using E, S_0, d_0 to represent the modulus, cross-section area, and total thickness respectively. When the sample was pressed with a force F, its thickness will change Δd, then F can be described as,

$$F = \frac{ES_0}{d_0}\Delta d \quad (9)$$

Thus, the ΔR_{con} and Δd can be as follows,

$$\Delta R_{\text{con}} = \frac{k}{[\frac{0.102ES_0}{d_0}\Delta d]^m} - R_{\text{con0}} \quad (10)$$

As strain ε is defined to be $\Delta d/d_0$, the relationship between ΔR_{con} and ε is as follows,

$$\Delta R_{\text{con}} \propto \frac{1}{\varepsilon^m} \quad (11)$$

To compare the slope with other two mechanisms, Equation 11 can be changed with mirror symmetry and translational symmetry, define ε' =1-ε, then the relationship between ΔR_{con} and ε' is as follows,

$$\Delta R_{\text{con}} \propto \frac{1}{(1-\varepsilon')^m} \qquad 12$$

It is usually a face type in the application, so $m = 1$.

Supplementary Note 3. Relations between Δ*J* and *ε* of F-N tunnelling effect.

According to the F-N tunnelling equation described below,

$$J = AE_d^2 \exp\left(B/E_d \right) \quad 13$$

where *A* and *B* are empirical constants ($A>0$, $B<0$). *J* is a function of E_d, which is the electric field between two neighbouring UHCS in this study.

E_d is defined as (*E* is the electric potential, *d* is the thickness),

$$E_d = \frac{E}{d} \quad 14$$

Δ*d* can be defined as follows,

$$d = d_0 - \Delta d \quad 15$$

Combine Equations 13-15 to get the relationship between *J* and Δ*d*,

$$\Delta J = A\left(\frac{E}{d_0(1-\frac{\Delta d}{d_0})}\right)^2 \exp\left[\frac{Bd_0(1-\frac{\Delta d}{d_0})}{E}\right] - J_0 \quad 16$$

As strain *ε* is defined to be $\Delta d/d_0$, the relationship between Δ*J* and *ε* is as follows (A>0 and B<0),

$$\Delta J \propto \left(\frac{1}{1-\varepsilon}\right)^2 \exp\left[\frac{-(1-\varepsilon)}{1}\right] \quad 17$$

Supplementary Note 4. Advantages of preloading for composite pressure sensors.

There are two reasons why a preloading process is needed in our manufacturing process: First, the sensing film and the electrodes are fabricated independently, so a preloading force is beneficial for ensuring good contact between the electrodes and the sensing film. Secondly, the sensor film is basically a polymer composite and it is widely recognized that the filler concentration in a polymer composite may vary from sample to sample even within the same batch during manufacturing. Given that the sensitivity of the composite film is highly dependent on the filler concentration, this variation in filler concentration may lead to an inconsistent sensitivity of the produced sensors. Therefore, in the fabrication process, we reduced the concentration slightly below the optimum concentration to avoid the inconsistency in sensor performance. Furthermore, by preloading process, the filler concentration can be tuned to make sure every sensor has the same sensitivity. Therefore, the preloading process is helpful in the sensor's performance and reliability. The preloading force can be controlled in the packaging process or tuned by the user before measurement. Of course, this may add an additional step in practical applications.

Supplementary Note 5. Basic requirements of the pressure sensing film for injection application in *in vivo*.

There are three prerequisites for the PDMS-based thin-film pressure sensors to be used in the implantable area.

First, the film should be flexible and thin enough to ensure a large sensing area after self-unfolding. A sensor array film should be large enough to cover the target organ in practical applications. In order to facilitate the implantation of the sensor array, it can be folded into a small part and be injected to the body. For example, to insert a 10 × 10 mm sensing film into a needle with diameter of 1.54 mm, the film should be folded at least for 5 times. To achieve the 5 times folding, the film should be thinner than 49.7 μm that can be calculated by Equation 18[50].

$$W = \pi t 2^{\frac{3}{2}(n-1)} \tag{18}$$

where W is the width of a square piece of film with a thickness of t, and n is the desired number of folds to be carried out along alternate directions. Our sensing film is 20 μm thick and flexible, which allows sufficient folding before inserting into the syringe needle.

Secondly, the film should have the ability to unfolded with no damage after being folded and injected. Most flexible sensors with ultra-high sensitivity based on micro/nano structure usually cannot be bended for 180° for multiple times, which will damage these structures. Additionally, the injected sensor should be able to unfold in seconds. When immersed in water, our folded sensor quickly unfolded within 9 s (Fig. 3a and Supplementary Movie 2), demonstrating its potential for injection into the body and *in vivo* self-unfolding to support large-area detection.

Thirdly, the implantable sensor should overcome the *in vivo* isostatic pressure. The isostatic pressure would greatly reduce the sensitivity of sensors while the *in vivo* application usually

needs high sensitivity. Our UHCS-PDMS keeps a 0.1 to 1 kPa^{-1} sensitivity under a 20 cm depth PBS solution which may satisfy some *in vivo* applications.

Supplementary Note 6. Establish the relationship between the current density, the external electrical field and three fabrication parameters (c_{UHCS}, d_{UHCS}, l_{spine}) in the UHCS-PDMS system.

The known parameters based on experimental measurement are as follows:

Average length of spines: $l_{\mathrm{spine}} = 80$ nm; tap density of UHCS: $\rho_{\mathrm{UHCS}} = 0.10188\ \mathrm{g \cdot cm^{-3}} = 0.10188\times10^{-21}\ \mathrm{g{\cdot}nm^{-3}}$; density of PDMS: $\rho_{\mathrm{PDMS}}=1.03\ \mathrm{g{\cdot}cm^{-3}}=1.03\times10^{-21}\ \mathrm{g{\cdot}nm^{-3}}$; thickness of the film: $h = 20$ μm; the test voltage $E= 1$ V.

The variables include:

Diameter of a UHCS (d_{UHCS}, nm), radius of a UHCS (r_{UHCS}, nm), mass of a UHCS (m_{UHCS}); radius of cylinder unit: Model 1 ($r_{\mathrm{cylinder1}}$, nm), Model 2 ($r_{\mathrm{cylinder2}}$, nm), Model 3 ($r_{\mathrm{cylinder3}}$, nm); diameter of cylinder unit: Model 1 ($d_{\mathrm{cylinder1}}$, nm), Model 2 ($d_{\mathrm{cylinder2}}$, nm), Model 3 ($d_{\mathrm{cylinder3}}$, nm); concentration of UHCS (c_{UHCS}, $\mathrm{g{\cdot}nm^{-3}}$).

Model 1. An upper boundary model is obtained when the neighbouring spheres are in direct contact with each other no matter how they move, i.e. the film is always conductive.

As shown in Supplementary Figure 15a, we first consider the one-dimensional situation where spheres (suppose the number of spheres is (n+1)) without spines are evenly distributed in a line with a fixed length (h). The inter-sphere distance can be easily calculated from the formula (h/(n+1)). Once the inter-sphere distance equals to the diameter of UHCS (d_{UHCS}), all spheres are contacted with their neighbours, and the number of spheres, which we define as (n_0+1), is the maximum number of spheres that this line can accommodate. Then, we consider the situation of spheres with spines: Because of the spiky structure, every sphere could be moved in its horizontal plane at a certain range while still keeping in contact with their neighbours by spines.

Supplementary Figure 15b shows two adjacent spheres at a critical position where spheres A and B can just contact with each other. By drawing a circle with point E as the centre and CE as the radius, a round area is formed. If the projection of all the sphere centres on the horizontal plane falls within this area, they will be in contact with their neighbours no matter how they moved horizontally. We define CE as the effective radius (r_{er}), thus a "cylinder 1 (Supplementary Figure 15c)" can be built with a radius of ($r_{\mathrm{cylinder1}}=r_{\mathrm{er}}+r_{\mathrm{UHCS}}+l_{\mathrm{spine}}$), in which all the ($n_0$+1) spheres will always be in contact with each other.

If spheres with a diameter of 600 nm are used, AB and AC are the radii of a hollow sphere (300 nm). BE is the spine length (80 nm). So, the effective radius can be calculated as follows:

$$r_{\mathrm{er}} = CE = \sqrt{l_{\mathrm{spine}}^2 + 2l_{\mathrm{spine}} r_{\mathrm{UHCS}}} = 233.2nm \qquad 19$$

The concentration of spheres in the cylinder 1 of Supplementary Figure 15c can be calculated as

$$c_{\mathrm{UHCS}} = \frac{m_{\mathrm{UHCSs}}}{V_{\mathrm{cylinder1}}} = \frac{(n+1)\,m_{\mathrm{UHCS}}}{\pi r_{\mathrm{cylinder1}}^2 h} = \frac{(n+1)\,\rho_{\mathrm{UHCS}} V_{\mathrm{UHCS}}}{\pi r_{\mathrm{cylinder1}}^2 h} = \frac{(n+1)\,\rho_{\mathrm{UHCS}} \pi d_{\mathrm{UHCS}}^3}{6\pi r_{\mathrm{cylinder1}}^2 h} \qquad 20$$

Where m_{UHCSs} is the total mass of all UHCS in a cylinder 1 unit, m_{UHCS} is the mass of one hollow sphere. Then,

$$n+1 = \frac{6 r_{\mathrm{cylinder1}}^2 h c_{\mathrm{UHCS}}}{\rho_{\mathrm{UHCS}} d_{\mathrm{UHCS}}^3} \qquad 21$$

As mentioned previously, (n_0+1) is the maximum number of spheres that a column can hold.

$$n_0 + 1 = \frac{h}{d_{\mathrm{UHCS}}} \qquad 22$$

Then,

$$n+1 < \frac{h}{d_{\mathrm{UHCS}}} \qquad 23$$

Thus,

$$c_{\text{UHCS}} < \frac{d_{\text{UHCS}}^{2}\rho_{\text{UHCS}}}{6r_{\text{cylinder1}}^{2}} \qquad 24$$

Model 2. Combination of the tunnelling distance with Model 1.

According to the F-N tunnelling equation described below,

$$J = \frac{q^2 E_d^2}{16\pi^2 \hbar \Phi_d} \exp\left[-\frac{4\left(2qm^*\right)^{1/2} \Phi_d^{3/2}}{3\hbar E_d} \right] = A E_d^2 \exp\left(B/E_d \right) \qquad 25$$

To estimate the unknown coefficients A and B, we fabricated two pure PDMS samples coated on HOPG (Highly Oriented Pyrolytic Graphite) to mimic the situation in the UHCS-PDMS-UHCS system. The measurement (Supplementary Figure 16a) was performed under an applied voltage of 10 V and the testing area was 1.3 cm^2. Sample 1 with a thickness of 460 nm shows an average current of 0.000054 A; Sample 2 with a thickness of 290 nm shows an average current of 0.006291 A.

Coefficients A and B can be obtained as follows:

$$A = 3.3292\times10^{-11}\ \text{A/V}^2,\quad B = -2.29947\times10^{6}\ \text{V/cm}$$

Even if two adjacent spheres do not contact with each other, current can still go through based on the F-N tunnelling effect. A variable t_d is introduced to taking the tunnelling effect into consideration. t_d is defined as the nearest distance between two adjacent spheres when they are not in direct contact. Thus, $t_d/2$ could be regarded as the extension of the radius with UHCS. In this situation, the cylinder diameter $d_{\text{cylinder2}}$ is increased compared with cylinder 1, and considering that the t_d is in the range of 0-30 nm of 600 nm diameter spheres, here we may use the following expression to calculate the diameter of cylinder1 with a parameter t_d.

$$d_{\text{cylinder1}} = 2[\sqrt{l_{\text{spine}}^2 + l_{\text{spine}}(d_{\text{UHCS}} + t_d})+0.5(d_{\text{UHCS}}+t_d)+l_{\text{spine}}] \approx 2(\sqrt{l_{\text{spine}}^2 + l_{\text{spine}} d_{\text{UHCS}}} + 0.5(d_{\text{UHCS}}+t_d)+l_{\text{spine}}) \quad 26$$

To simplify the following calculation, and this estimation makes a variation less than 0.8%, thus, $d_{\text{cylinder2}}$ can be calculated as follows,

$$d_{\text{cylinder2}} = d_{\text{cylinder1}} + t_d \approx 2\left(\sqrt{l_{\text{spine}}^2 + l_{\text{spine}} d_{\text{UHCS}}} + 0.5 d_{\text{UHCS}} + l_{\text{spine}}\right) + t_d \quad 27$$

The volume ($V_{\text{cylinder2}}$) of the cylinder can be written as follows:

$$V_{\text{cylinder2}} = \frac{\pi d_{\text{cylinder2}}^2}{4} h \quad 28$$

The numbers ($n_{\text{cylinder2}}$) of UHCS in this cylinder can be written as follows:

$$n_{\text{cylinder2}} = \frac{h}{d_{\text{UHCS}} + t_d} \quad 29$$

The mass ($m_{\text{cyclinder2}}$) of UHCS in one cylinder can be written as,

$$m_{\text{cylinder2}} = n_{\text{cylinder2}} \rho_{\text{UHCS}} V_{\text{UHCS}} \quad 30$$

Combined with Equations 7- 10, the c_{UHCS} of UHCS should be written by,

$$c_{\text{UHCS}} = \frac{\frac{h}{d_{\text{UHCS}} + t_d} \rho_{\text{UHCS}} \pi d_{\text{UHCS}}^3}{\frac{\pi\left(d_{\text{cylinder1}} + t_d\right)^2}{4} h \times 6} = \frac{2\rho_{\text{UHCS}} d_{\text{UHCS}}^3}{3\left(d_{\text{UHCS}} + t_d\right)\left(d_{\text{cylinder1}} + t_d\right)^2} \quad 31$$

By changing Equation 31 into a cubic equation of t_d,

$$t_d^3 + \left(2d_{\text{cylinder1}} + d_{\text{UHCS}}\right)t_d^2 + d_{\text{cylinder1}}\left(d_{\text{cylinder1}} + 2d_{\text{UHCS}}\right)t_d + d_{\text{UHCS}} d_{\text{cylinder1}}^2 - \frac{2\rho_{\text{UHCS}} d_{\text{UHCS}}^3}{3c_{\text{UHCS}}} = 0 \quad 32$$

By putting $d_{\text{cylinder1}}$ into Equation 32, we could deduce the relationship between t_d, l_{spine}, d_{UHCS}, and c_{UHCS} as follows:

$$t_d^3 + \left[2\left(4\sqrt{l_{\text{spine}}^2 + l_{\text{spine}} d_{\text{UHCS}}} + d_{\text{UHCS}} + 2l_{\text{spine}}\right) + d_{\text{UHCS}} \right] t_d^2 +$$
$$\left[\begin{array}{l} \left(4\sqrt{l_{\text{spine}}^2 + l_{\text{spine}} d_{\text{UHCS}}} + d_{\text{UHCS}} + 2l_{\text{spine}}\right)^2 \\ +2d_{\text{UHCS}}\left(4\sqrt{l_{\text{spine}}^2 + l_{\text{spine}} d_{\text{UHCS}}} + d_{\text{UHCS}} + 2l_{\text{spine}}\right) \end{array} \right] t_d +$$
$$d_{\text{UHCS}}\left(4\sqrt{l_{\text{spine}}^2 + l_{\text{spine}} d_{\text{UHCS}}} + d_{\text{UHCS}} + 2l_{\text{spine}}\right)^2 - \frac{2\rho_{\text{UHCS}} d_{\text{UHCS}}^3}{3c_{\text{UHCS}}} = 0 \qquad 33$$

To solve the cubic Equation 32, we define x=t_d. Thus, this equation can be reduced as,

$$ax^3 + bx^2 + cx + d = 0 \qquad 34$$

From Equations 32 and 34,

$$\begin{cases} a = 1 \\ b = 2d_{\text{cylinder1}} + d_{\text{UHCS}} \\ c = d_{\text{cylinder1}}\left(d_{\text{cylinder1}} + 2d_{\text{UHCS}}\right) \\ d = d_{\text{UHCS}} d_{\text{cylinder1}}^2 - \dfrac{2\rho_{\text{UHCS}} d_{\text{UHCS}}^3}{3c_{\text{UHCS}}} \end{cases} \qquad 35$$

Assume,

$$x = y - \frac{b}{3a} \qquad 36$$

We can change Equation 34 into,

$$y^3 + py + q = 0 \qquad 37$$

In which p and q can be calculated as follows from Equation 36 and Equation 37,

$$\begin{cases} p = \dfrac{3c - b^2}{3} \\ q = d - \dfrac{9bc - 2b^3}{27} \end{cases} \qquad 38$$

Bring Equation 35 into Equation 38,

$$\begin{cases} p = \dfrac{2d_{\text{cylinder1}}\left(d_{\text{cylinder1}} + 2d_{\text{UHCS}}\right) - \left(2d_{\text{cylinder1}} + d_{\text{UHCS}}\right)^2}{3} \\ q = d_{\text{UHCS}} d_{\text{cylinder1}}^2 - \dfrac{2\rho_{\text{UHCS}} d_{\text{UHCS}}^3}{3c_{\text{UHCS}}} - \dfrac{9\left(2d_{\text{cylinder1}} + d_{\text{UHCS}}\right) d_{\text{cylinder1}}\left(d_{\text{cylinder1}} + 2d_{\text{UHCS}}\right) - 2\left(2d_{\text{cylinder1}} + d_{\text{UHCS}}\right)^3}{27} \end{cases} \quad 39$$

Known from Cardano's Formula, $\Delta = \left(\frac{q}{2}\right)^2 + \left(\frac{p}{3}\right)^3$

As $\Delta < 0$, there are three unequal real roots, so we further replace p and q with parameters r and θ, where

$$\begin{cases} r = \sqrt{-\left(\dfrac{p}{3}\right)^3} \\ \theta = \dfrac{1}{3}\arccos\left(-\dfrac{q}{2r}\right) \end{cases} \quad 40$$

Then the solutions to Equation 37 are

$$\begin{cases} y_1 = 2\sqrt[3]{r}\cos\theta \\ y_2 = 2\sqrt[3]{r}\cos\left(\theta + \dfrac{2}{3}\pi\right) \\ y_3 = 2\sqrt[3]{r}\cos\left(\theta + \dfrac{4}{3}\pi\right) \end{cases} \quad 41$$

$$t_d = x_i = y_i - \frac{b}{3a}, (i = 1, 2, 3) \quad 42$$

As there are three real roots, in this study, t_d is the distance between two spines, so we can choose the root $0 < x_i < 600$ as the root for t_d.

On the other hand, according to the electric-field characteristics on the point of a conical conductor[51], the electric potential difference is,

$$\Delta U \approx C r^{\lambda} \quad 43$$

where ΔU is the electric potential difference, C is a constant determined by the electric and geometric parameters, r is the distance, λ is a constant determined by geometric parameters.

As shown in Supplementary Figure 16b, A and B are located at the bottom of two spines of two adjacent UHCS. The electrical potential of A and B is defined as $U_A=u_0$, $U_B=0$ respectively. The distance r between A and B is approximately ($2l_{\text{spine}}+t_d$). Since the surface of UHCS is an equipotential surface, according to the voltage division principle, the electric potential difference between A and B is,

$$U_B - U_A = 0 - u_0 = \frac{E}{n_{\text{cylinder2}}} \approx C(2l_{\text{spine}} + t_d)^{\lambda} \qquad 44$$

Thus, C can be calculated as,

$$C = \frac{E}{n_{\text{cylinder2}}}(2l_{\text{spine}} + t_d)^{-\lambda} \qquad 45$$

The parameter λ can be calculated as follows[51]

$$\lambda = [2\ln(\frac{2}{\pi - \beta})]^{-1} \approx 0.163209 \qquad 46$$

where β is the angle between the central line and the surface of the spine as shown in Supplementary Figure 16c.

Taken Equations 44-46 together, the electric intensity E_d between two neighbouring spines can be calculated as

$$E_d = -\frac{\partial U}{\partial r}\Big|_{r=t_d} \approx -\lambda C t_d^{\lambda-1} = -\lambda \frac{E}{n_{\text{cylinder2}}}(2l_{\text{spine}} + t_d)^{-\lambda} t_d^{\lambda-1} \qquad 47$$

Taking t_d into Equation 47, we can calculate E_d as a function of the three fabrication parameters of the sensing film (i.e. d_{UHCS}, l_{spine}, c_{UHCS}) within a fixed the external field. Therefore, by putting E_d into Equation 25, we can calculate the relationship between current density and the

concentration and diameter of UHCS (with given spine length and external field), as shown in Fig. 4b of the main text.

Supplementary Note 7. Calculation of the minimum detecting area statistically.

Model 3. Establish the probability model to calculate the minimum concentration of UHCS.

As shown in Supplementary Figure 17, we define a new cylinder unit in Model 3. Suppose one sphere already exists in the centre when the second sphere is added to the cylinder. When the projection of the centre of the second sphere drops into the green circle, the radius of which is $2r_{\mathrm{er}}$, the probability of the two UHCSs in contact with each other is 100%. Also, we define a yellow circle to double the area of the green circle. The radius of the yellow circle will be,

$$r_{\mathrm{yellow}} = \sqrt{8r_{\mathrm{er}}^2} \approx 659.5892\mathrm{nm} \quad 48$$

Thus, when the centre of the second sphere drops in the yellow circle, the probability of the two UHCS contacting each other is 50%. All the UHCSs whose centre drops on the yellow circle will form the big black section, which defines the border of cylinder 3, as shown in Supplementary Figure 17. The radius of cylinder 3 is,

$$r_{\mathrm{cylinder3}} = r_{\mathrm{yellow}} + r_{\mathrm{UHCS}} + l_{\mathrm{spine}} \approx 1039.5892\mathrm{nm} \quad 49$$

When (n_0+1) UHCSs are placed in cylinder 3, where $n_0+1=h/d_{\mathrm{UHCS}}$, the probability of two neighbouring UHCSs contacting with each other is 1/2, and for the whole column to form a conductive pathway is $(1/2)^n$, approaching 0. Therefore, this situation is used as the lower boundary for c_{UHCS}.

$$c_{UHCS} = \frac{d_{UHCS}^2 \rho_{UHCS}}{6r_{cylinder3}^2} \quad 50$$

then

$$c_{UHCS} = \frac{\mathrm{d}_{\mathrm{UHCS}}^2 0.10188}{6\left[\sqrt{8\left(l_{\mathrm{spine}}^2 + l_{\mathrm{spine}} d_{\mathrm{UHCS}}\right)} + 0.5d_{\mathrm{UHCS}} + l_{\mathrm{spine}}\right]^2} \quad 51$$

Model 4. Calculation of the minimum detectable sensing area at a high probability

For a basic unit in cylinder 3 with a height of d_{UHCS}, two UHCSs are added and the probability of contacting each other is 1/2. To make the cylinder conductive, more UHCSs can be added to the unit. With a content of c_{UHCS}, the spheres number (k+1) in this basic unit is defined, and k can be calculated by the following formula.

$$k=\frac{(n+1)-1}{(n_0+1)-1}=\frac{\dfrac{6r_{\mathrm{cylinder3}}^2 c_{\mathrm{UHCS}}\pi h}{\rho_{\mathrm{UHCS}}\pi d_{\mathrm{UHCS}}^3}-1}{\dfrac{h}{d_{UHCS}}-1}=\frac{6r_{\mathrm{cylinder3}}^2 c_{\mathrm{UHCS}} h-\rho_{\mathrm{UHCS}} d_{\mathrm{UHCS}}^3}{\rho_{\mathrm{UHCS}} d_{\mathrm{UHCS}}^2 (h-d_{UHCS})} \qquad 52$$

We define P_0 as the probability when two UHCS cannot contact with each other in a basic unit in cylinder 3 (shown in Supplementary Figure 18),

$$P_0=\left[\frac{S_{\mathrm{yellow}}-S_{\mathrm{green}}-(k-1)\pi r_{\mathrm{er}}^2}{S_{\mathrm{yellow}}}\right]^k \qquad 53$$

Where,

$$S_{\mathrm{green}}=\pi\left(2r_{\mathrm{er}}\right)^2, S_{\mathrm{yellow}}=\pi r_{\mathrm{double}}^2=8\pi r_{er}^2 \qquad 54$$

So,

$$P_0=\left(\frac{5-k}{8}\right)^k \qquad 55$$

The probability of a cylinder unit to be conductive is defined as P_1 (Supplementary Figure 18), thus

$$P_1=\left(1-P_0\right)^{n_0} \qquad 56$$

Define P_2 (Supplementary Figure 18) as the probability that an area that contains x cylinder units is not conductive. If the probability of this area to be conductive is 97 %, then,

$$P_2 = (1-P_1)^x = 1-97\% \quad 57$$

This equation can be rewritten as follows,

$$x = \frac{1}{\log_{0.03}(1-P_1)} = \frac{\ln 0.03}{\ln(1-P_1)} \quad 58$$

Put the parameter of c_{UHCS}=15 g • L^{-1}, d_{UHCS}=600 nm, l_{spine}=80 nm, h=20 μm, ρ_{UHCS}=101.88 g·L^{-1} into Equations (50-58), k=2.7031, P_0=0.034282, P_1=0.31262, then x could be calculated,

$$x = 9.354 \quad 59$$

Therefore, x cylinder units with a radius of $r_{\text{cylinder3}}$ could work as the minimum detecting area of a large probability (>97 %) to be conductive and sensitive. The minimum detection $S_{\min}$ and minimum side length $a_{\min}$ can be calculated as,

$$S_{\min} = x\pi r_{\text{cylinder3}}^2 = 31.7\mu\text{m}^2 \quad 60$$

$$a_{\min} = \sqrt{S_{\min}} = 5.63\mu\text{m} \quad 61$$

Also, the thickness of electrodes in this study is about 35 nm (5 nm Cr and 30 nm Au), two adjacent electrodes be separated with a distance of $d_{\min}$ nm could form a cube (35 × 5630 × $d_{\min}$ nm^3), in this cube, h=$d_{\min}$, thus k^*, P^*_0, could be calculated by Equations 50, 52. As the cross-sectional area 35 × 5630 nm^2<< $S_{\text{cylinder3}}$, P_1 is the conductive percentage of a Cylinder3 unit. Its conductive percentage (P^*_3) should be less than 3% to achieve a statistically insulation,

$$P_3^* = \left[\frac{35\times 5630}{S_{\text{cylinder3}}}(1-P_0^*)\right]^{\frac{d_{\min}}{600}} < 3\% \quad 62$$

$$d_{\min} > 435\text{nm} \quad 63$$

From the above, a theoretical density n_{den} can be calculated using the minimum area and minimum pitch size,

$$n_{\text{den}} = \frac{10^{14}}{\left(a_{\min} + d_{\min}\right)^2} = 2718557 cm^{-2} \tag{64}$$

Supplementary References

1. Sheng L*, et al.* , Bubble-decorated honeycomb-like graphene film as ultrahigh sensitivity pressure sensors. *Adv. Funct. Mater.* **25**, 6545-6551 (2015).

2. Pan L*, et al.* , An ultra-sensitive resistive pressure sensor based on hollow-sphere microstructure induced elasticity in conducting polymer film. *Nat. commun.* **5**, 3002 (2014).

3. Choong CL*, et al.* , Highly stretchable resistive pressure sensors using a conductive elastomeric composite on a micropyramid array. *Adv. Mater.* **26**, 3451-3458 (2014).

4. Su B*, et al.* , Mimosa-inspired design of a flexible pressure sensor with touch sensitivity. *Small* **11**, 1886-1891 (2015).

5. Jian M*, et al.* , Flexible and highly sensitive pressure sensors based on bionic hierarchical structures. *Adv. Funct. Mater.* **27**, 1606066 (2017).

6. Tao LQ*, et al.* , Graphene-paper pressure sensor for detecting human motions. *ACS Nano.* **11**, 8790-8795 (2017).

7. Shao Q*, et al.* , High-performance and tailorable pressure sensor based on ultrathin conductive polymer film. *Small* **10**, 1466-1472 (2014).

8. Hou C*, et al.* , Highly conductive, flexible, and compressible all-graphene passive electronic skin for sensing human touch. *Adv. Mater.* **26**, 5018-5024 (2014).

9. Park J*, et al.* , Giant tunnelling piezoresistance of composite elastomers with interlocked microdome arrays for ultrasensitive and multimodal electronic skins. *ACS Nano.* **8**, 4689-4697 (2014).

10. Zhang J*, et al.* Ultra-sensitive transparent and stretchable pressure sensor with single electrode. 2016 IEEE 29th International Conference on Micro Electro Mechanical Systems (MEMS), Shanghai, 2016, pp. 173-176.

11. Bae GY*, et al.* , Linearly and highly pressure-sensitive electronic skin based on a bioinspired hierarchical structural array. *Adv. Mater.* **28**, 5300-5306 (2016).

12. Schwartz G*, et al.* , Flexible polymer transistors with high pressure sensitivity for application in electronic skin and health monitoring. *Nat. Commun.* **4**, 1859 (2013).

13. Zhou X, *et al.* , Flexible and highly sensitive pressure sensors based on microstructured carbon nanowalls electrodes. *Nanomaterials-Basel.* **9(4)**, 496 (2019).

14. Fu X, Dong H, Zhen Y, Hu W, Solution-processed large-area nanocrystal arrays of metal–organic frameworks as wearable, ultrasensitive, electronic skin for health monitoring. *Small* **11**, 3351-3356 (2015).

15. Zhu B*, et al.* , Microstructured graphene arrays for highly sensitive flexible tactile sensors. *Small* **10**, 3625-3631 (2014).

16. Wu Y*, et al.* , A skin-inspired tactile sensor for smart prosthetics. *Sci. Robot.* **3**, eaat0429 (2018).

17. Wu W, Wen X, Wang ZL, Taxel-addressable matrix of vertical-nanowire piezotronic transistors for active and adaptive tactile imaging. *Science* **340**, 952-957 (2013).

18. Wang X*, et al.* , Electronic skin: silk-molded flexible, ultrasensitive, and highly stable electronic skin for monitoring human physiological signals. *Adv. Mater.* **26**, 1309-1309 (2014).

19. Park S*, et al.* , Stretchable energy-harvesting tactile electronic skin capable of differentiating multiple mechanical stimuli modes. *Adv. Mater.* **26**, 7324-7332 (2014).

20. Wang C*, et al.* , User-interactive electronic skin for instantaneous pressure visualization. *Nat. Mater.* **12**, 899 (2013).

21. Gong S*, et al.* , A wearable and highly sensitive pressure sensor with ultrathin gold nanowires. *Nat. Commun.* **5**, 3132 (2014).

22. Viry L*, et al.* , Flexible three-axial force sensor for soft and highly sensitive artificial touch. *Adv. Mater.* **26**, 2659-2664 (2014).

23. Huang CB*, et al.* , Molecule-graphene hybrid materials with tunable mechanoresponse: highly sensitive pressure sensors for health monitoring. *Adv. Mater.* **31**, e1804600 (2019).

24. Wan S*, et al.* , Graphene oxide as high-performance dielectric materials for capacitive pressure sensors. *Carbon* **114**, 209-216 (2017).

25. Mannsfeld SC*, et al.* , Highly sensitive flexible pressure sensors with microstructured rubber dielectric layers. *Nat. Mater.* **9**, 859 (2010).

26. Dong X*, et al.* , A linear and large-range pressure sensor based on a graphene/silver nanowires nanobiocomposites network and a hierarchical structural sponge. *Compos. Sci. Technol.* **155**, 108-116 (2018).

27. Lei Z*, et al.* , A bioinspired mineral hydrogel as a self-healable, mechanically adaptable ionic skin for highly sensitive pressure sensing. *Adv. Mater.* **29**, 1700321 (2017).

28. Sun Q*, et al.* , Transparent, low-power pressure sensor matrix based on coplanar-gate graphene transistors. *Adv. Mater.* **26**, 4735-4740 (2014).

29. Gao Y*, et al.* , Wearable microfluidic diaphragm pressure sensor for health and tactile touch monitoring. *Adv. Mater.* **29**, 1701985(2017).

30. Wu X*, et al.* , Large-area compliant, low-cost, and versatile pressure-sensing platform based on microcrack-designed carbon Black@ polyurethane sponge for human–machine interfacing. *Adv. Funct. Mater.* **26**, 6246-6256 (2016).

31. Tang Y*, et al.* , Manufacturable conducting rubber ambers and stretchable conductors from copper nanowire aerogel monoliths. *ACS Nano.* **8**, 5707-5714 (2014).

32. Tai Y, Lubineau G, Double-twisted conductive smart threads comprising a homogeneously and a gradient-coated thread for multidimensional flexible pressure-sensing devices. *Adv. Funct. Mater.* **26**, 4078-4084 (2016).

33. Pu X*, et al.* , Ultrastretchable, transparent triboelectric nanogenerator as electronic skin for biomechanical energy harvesting and tactile sensing. *Sci. Adv.* **3**, e1700015 (2017).

34. Zou Z*, et al.* , Rehealable, fully recyclable, and malleable electronic skin enabled by dynamic covalent thermoset nanocomposite. *Sci. Adv.* **4**, eaaq0508 (2018).

35. Johansson, R. S. & Vallbo, A. B. Tactile sensibility in the human hand: relative and absolute densities of four types of mechanoreceptive units in glabrous skin. *J. Physiol.* **286**, 283-300, (1979).

36. Wang, C. *et al.* User-interactive electronic skin for instantaneous pressure visualization. *Nat. Mater.* **12**, 899-904, (2013).

37. Lipomi, D. J. *et al.* Skin-like pressure and strain sensors based on transparent elastic films of carbon nanotubes. *Nat. Nanotechnol.* **6**, 788-792, (2011).

38. Ho, D. H. *et al.* Stretchable and multimodal all graphene electronic skin. *Adv. Mater.* **28**, 2601-2608, (2016).

39. Lee, K. Y. *et al.* Fully packaged self-powered triboelectric pressure sensor using hemispheres-array. *Adv. Energy. Mater.* **6**, (2016).

40. Boutry, C. M. *et al.* A sensitive and biodegradable pressure sensor array for cardiovascular monitoring. *Adv. Mater.* **27**, 6954-6961, (2015).

41. Yeom, C. *et al.* Large-area compliant tactile sensors using printed carbon nanotube active-matrix backplanes. *Adv. Mater.* **27**, 1561-1566, (2015).

42. Someya, T. *et al.* Conformable, flexible, large-area networks of pressure and thermal sensors with organic transistor active matrixes. *P. Natl. A. Sci.* **102**, 12321-12325, (2005).

43. Liu, M. *et al.* Large-area all-textile pressure sensors for monitoring human motion and physiological signals. *Adv. Mater.* **29**, (2017).

44. Zang, Y. *et al.* Flexible suspended gate organic thin-film transistors for ultra-sensitive pressure detection. *Nat. Commun.* **6**, 6269, (2015).

45. Dong, K. *et al.* A stretchable yarn embedded triboelectric nanogenerator as electronic skin for biomechanical energy harvesting and multifunctional pressure sensing. *Adv. Mater.* **30**, e1804944, (2018).

46. Tee, B. C. K. , Wang, C. , Allen, R. & Bao, Z. An electrically and mechanically self-healing composite with pressure- and flexion-sensitive properties for electronic skin applications. *Nat. Nanotechnol.* **7**, 825-832, (2012).

47. Tee, B. C. -K. *et al.* A skin-inspired organic digital mechanoreceptor. *Science* **350**, 313-316, (2015).

48. Rintoul, M. D. & Torquato, S. Precise determination of the critical threshold and exponents in a three-dimensional continuum percolation model. *J. Phys. A. Math. Gen.* **30,** L585-L592, (1997).

49. XU, J. & LI, K. The research on resistance of electrical contact. Electr. Eng. Mater. **1,** 10-13, (2011).

50. Gallivan, B. Paper folding theorem. https://en.wikipedia.org/wiki/Britney_Gallivan (2020).

51. Rong Y, The electric-field characteristics on the point of a conical conductor. *Coll. Phys.* **9**, 18-20, (2003).
