# Supplementary material for: Quantum effect-based flexible and transparent pressure sensors with ultrahigh sensitivity and sensing density
Source: Nat Commun. 2020 Jul 15;11:3529. doi: 10.1038/s41467-020-17298-y (PMC7363923; doi:10.1038/s41467-020-17298-y)
Supplement: Supplementary file 6 — Description of Additional Supplementary Files [file 41467_2020_17298_MOESM6_ESM.pdf]

Title: Supplementary Movie 1.

Description: Pressure test of the tiny sensor on a fingertip. A small piece of cotton dropped onto the tiny sensor and its resistance changed immediately to show its sensing ability.

Title: Supplementary Movie 2.

Description: Self-unfolding behaviour of the UHCS-PDMS. A UHCS-PDMS thin film that has been folded several times was put into a petri dish with deionized water. It shows immediate self-unfolding behaviour in 9 seconds.

Title: Supplementary Movie 3.

Description: Dynamic array test for UHCS-PDMS. A 64 × 64 passive sensing array on a 32 mm × 32 mm film sensor was tested to show its resolution, shape identify and dynamic sensing ability.
